# Supplementary material for: Merlin tumor suppressor function is regulated by PIP2-mediated dimerization
Source: PLoS One. 2023 Feb 21;18(2):e0281876. doi: 10.1371/journal.pone.0281876 (PMC9942953; doi:10.1371/journal.pone.0281876)
Supplement: S1 Fig — A). Merlin isoform dimerization reactions with Merlin isoform 1-NL: Merlin isoform 1-GFP, Merlin isoform 2-NL: Merlin isoform 1-GFP and Merlin isoform 2-NL: Merlin isoform 2-GFP. The data is a mean of triplicate binding reactions with standard deviation and expressed as a percentage of the Merlin isoform 1. B). Emission spectrum from 400 nm to 600 nm of Merlin isoform dimerization assays normalized to the 450 nm peak. (DOCX) [file pone.0281876.s001.docx]

## Supplemental Figure 1


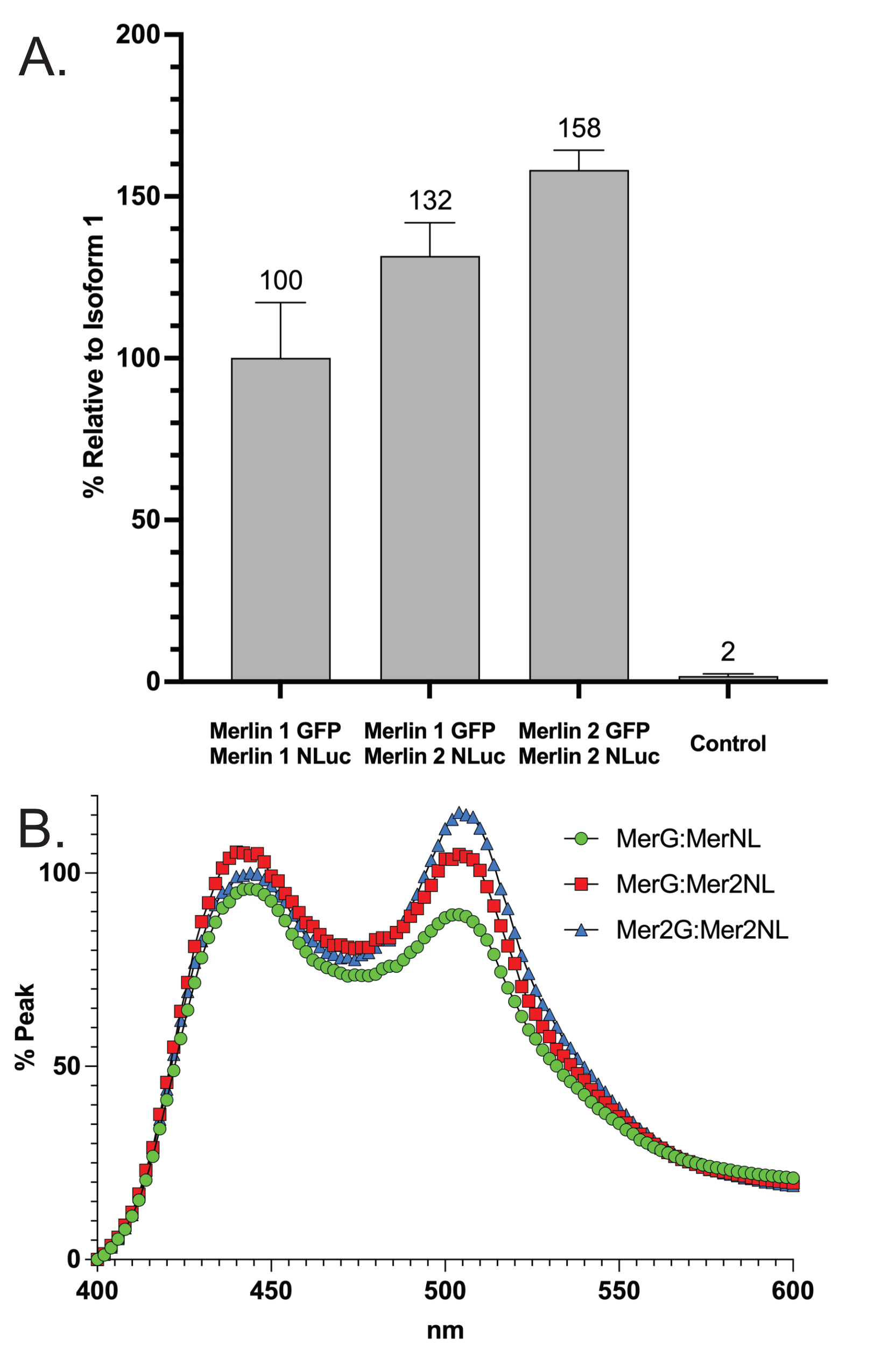


### Supplemental Figure 1.

A). Merlin isoform dimerization reactions with Merlin isoform 1-NL: Merlin isoform 1-GFP, Merlin isoform 2-NL: Merlin isoform 1-GFP and Merlin isoform 2-NL: Merlin isoform 2-GFP. The data is a mean of triplicate binding reactions with standard deviation and expressed as a percentage of the Merlin isoform 1.

B). Emission spectrum from 400 nm to 600 nm of Merlin isoform dimerization assays normalized to the 450 nm peak.
