# Supplementary material for: Merlin tumor suppressor function is regulated by PIP2-mediated dimerization
Source: PLoS One. 2023 Feb 21;18(2):e0281876. doi: 10.1371/journal.pone.0281876 (PMC9942953; doi:10.1371/journal.pone.0281876)
Supplement: S3 Fig — A). BRET assays showing emission spectrum from 400 nm to 600 nm from dimerization assays for combinations of the Merlin phosphorylation mutants S518A:S518A, S518A:S518D and S518D:S518D normalized to the 450 nm peak. B). BRET assays showing emission spectrum from 400 nm to 600 nm from dimerization assays for combinations of the Merlin conformation mutants AR:AR, AR:ΔEL and ΔEL:ΔEL normalized to the 450 nm peak. (DOCX) [file pone.0281876.s003.docx]

## Supplemental Figure 3


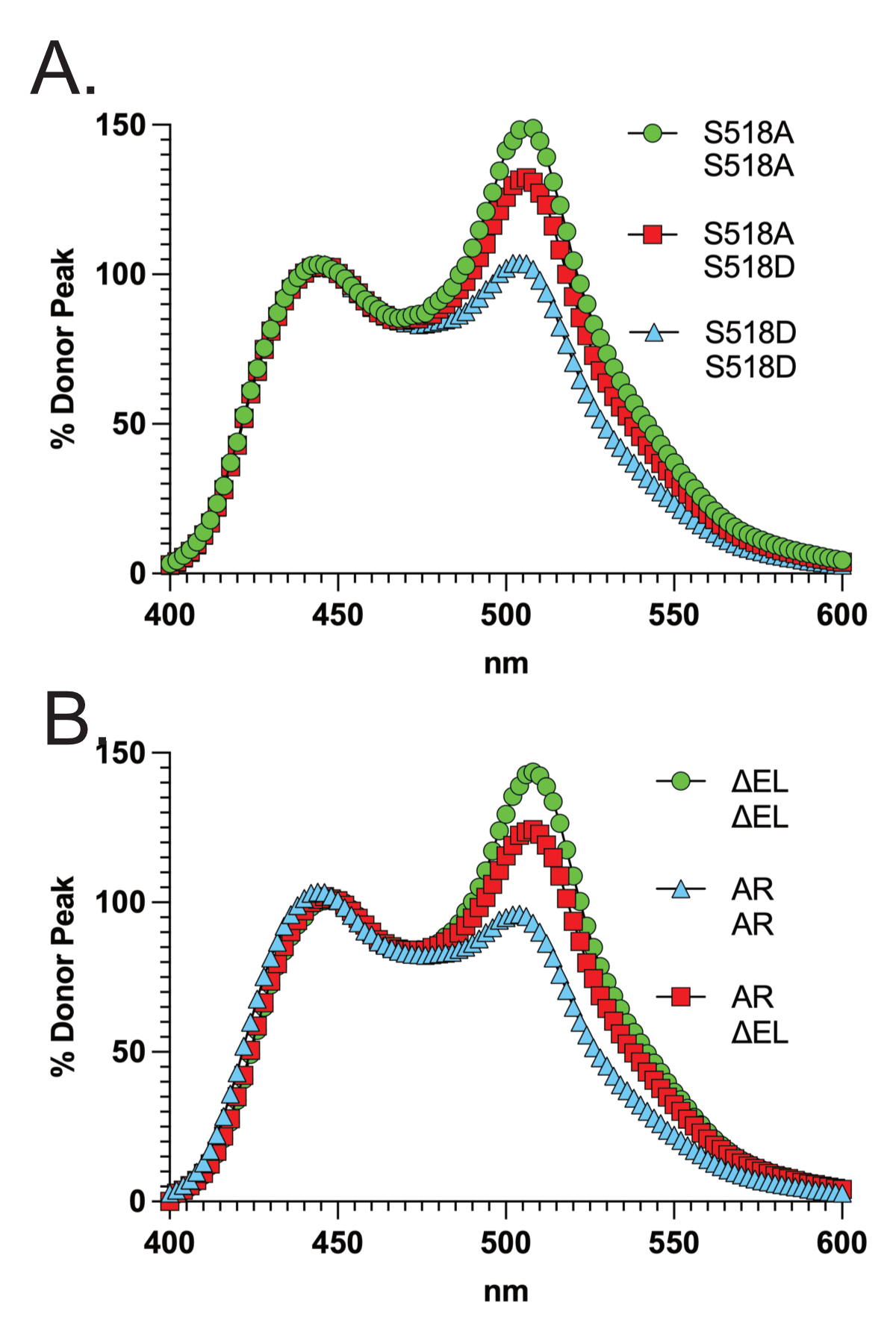


A). BRET assays showing emission spectrum from 400 nm to 600 nm from dimerization assays for combinations of the Merlin phosphorylation mutants S518A:S518A, S518A:S518D and S518D:S518D normalized to the 450 nm peak.

B). BRET assays showing emission spectrum from 400 nm to 600 nm from dimerization assays for combinations of the Merlin conformation mutants AR:AR, AR:ΔEL and ΔEL:ΔEL normalized to the 450 nm peak.
