# Supplementary material for: Merlin tumor suppressor function is regulated by PIP2-mediated dimerization
Source: PLoS One. 2023 Feb 21;18(2):e0281876. doi: 10.1371/journal.pone.0281876 (PMC9942953; doi:10.1371/journal.pone.0281876)
Supplement: S4 Fig — A listing of the primers, plasmids and the amino acid sequences of the recombinant Merlin proteins used in this manuscript. (DOCX) [file pone.0281876.s004.docx]

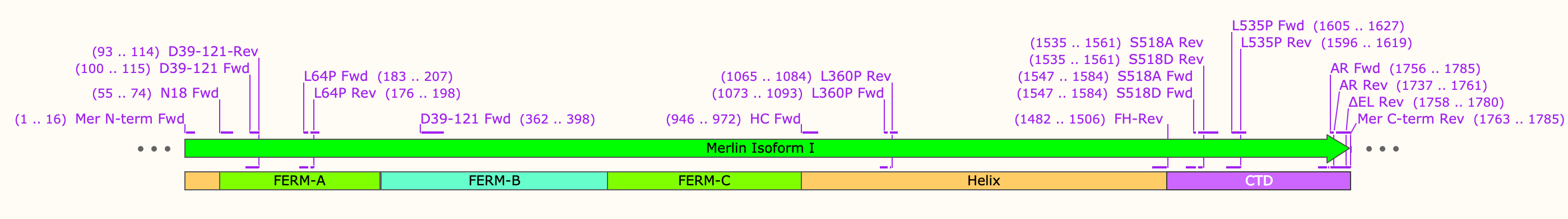


**Full Length Merlin**

Mer N-term Fwd ATGGCCGGAGCCATCG

Mer C-term Rev GAGCTCTTCAAAGAAGGCCACTC

**Merlin Mutants**

N18 Fwd ATGCCCAAGACGTTCACCGTGAG

Mer C-term Rev GAGCTCTTCAAAGAAGGCCACTC

Δ39-121 Fwd GAGTTCAATTGCGAGAGGTAAAGAAGCAGATTTTAGATGAAAAGATCTACTG

Δ39-121-Rev CTCGCAATTGAACTCCATCTCG

L64P Fwd CTTTGGACCGCAGTACACAATCAAG

L64P Rev GTACTGCGGTCCAAAGAACCAGG

HC Fwd TTGGAAGTTCAGCAGATGAAAGCCCAG

Mer C-term Rev GAGCTCTTCAAAGAAGGCCACTC

L360P Fwd GGAGGCCGCTGCAGATGAAAG

L360P Rev GCAGCGGCCTCCTCTCCAAC

Mer N-term Fwd ATGGCCGGAGCCATCG

FH-Rev ACCAATGAGGTTGAAGCTTGGTATG

S518D Fwd GGCTTGACATGGAGATAGAGAAAGAAAAAGTGGAATAC

S518D Rev TCTCCATGTCAAGCCGCTTCATGTCAG

S518A Fwd GGCTTGCCATGGAGATAGAGAAAGAAAAAGTGGAATAC

S518A Rev TCTCCATGGCAAGCCGCTTCATGTCAG

L535P Fwd CCGCAGGAGCAGCTCAATGAACTCA

L535P Rev TTGAGCTGCTCCTGCGGATGCTTG

AR Fwd TGGAAGTCCAAAGTGGCCTTCTTTGAAGAGCTC

AR Rev TTTGGACTTCCAGCTCTGCAAGGTGAGC

Mer N-term Fwd ATGGCCGGAGCCATCG

∆EL Rev CACCATGGTGGATCCTTCAAAGAAGGCCACTCGGGAC

N20 Fwd AGGAAGCAACCCAAGGGATCCACCATGGTGAGCAAG

N20 Rev CTTGGGTTGCTTCCTCTTGAGAG

HC Fwd ATGTTGGAAGTTCAGCAGATGAAAGCCCAG

Mer C-term Rev GAGCTCTTCAAAGAAGGCCACTC

HC Fwd ATGTTGGAAGTTCAGCAGATGAAAGCCCAG

delCTD Fwd GCTTCAACCTCATTGGTGGATCCACCATGGTGAGCAAG

**Angiomotin**

AMOTp130-5' AGAAATTCTGAcGAACAGCCAAGTG

AMOTp130-3' GATGAGATATTCCACCATCTCTGCATCAG

**NanoLuc**

NanoLuc Insert Fwd GTCTTCACACTCGAAGATTTCGTTG

NanoLuc Insert Rev CAGCTCGTCCATGCCCGCCAGAATGCGTTCGC

**Vector Cloning Primers**

ST GFP/NL Vec Fwd TAAgcggccgcgactctag

ST-GFP/NL Vec Rev CTTGTACAGCTCGTCCATGCC


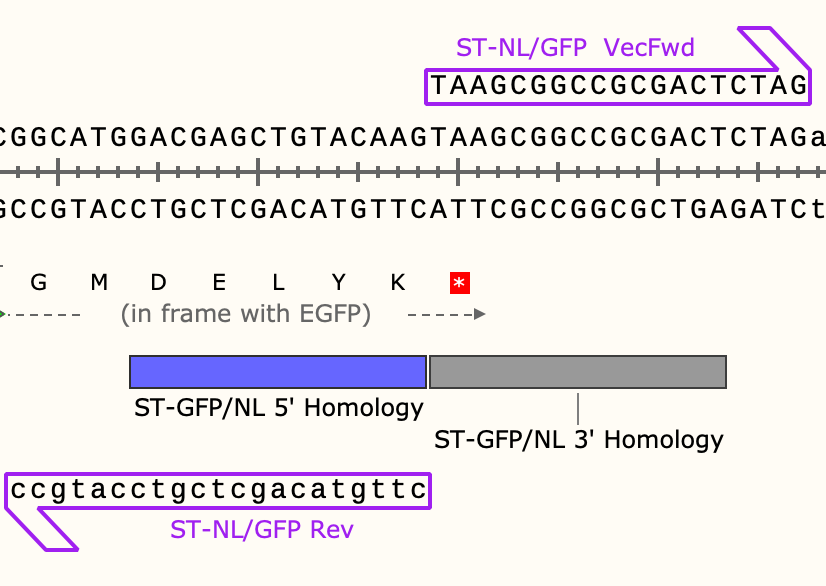


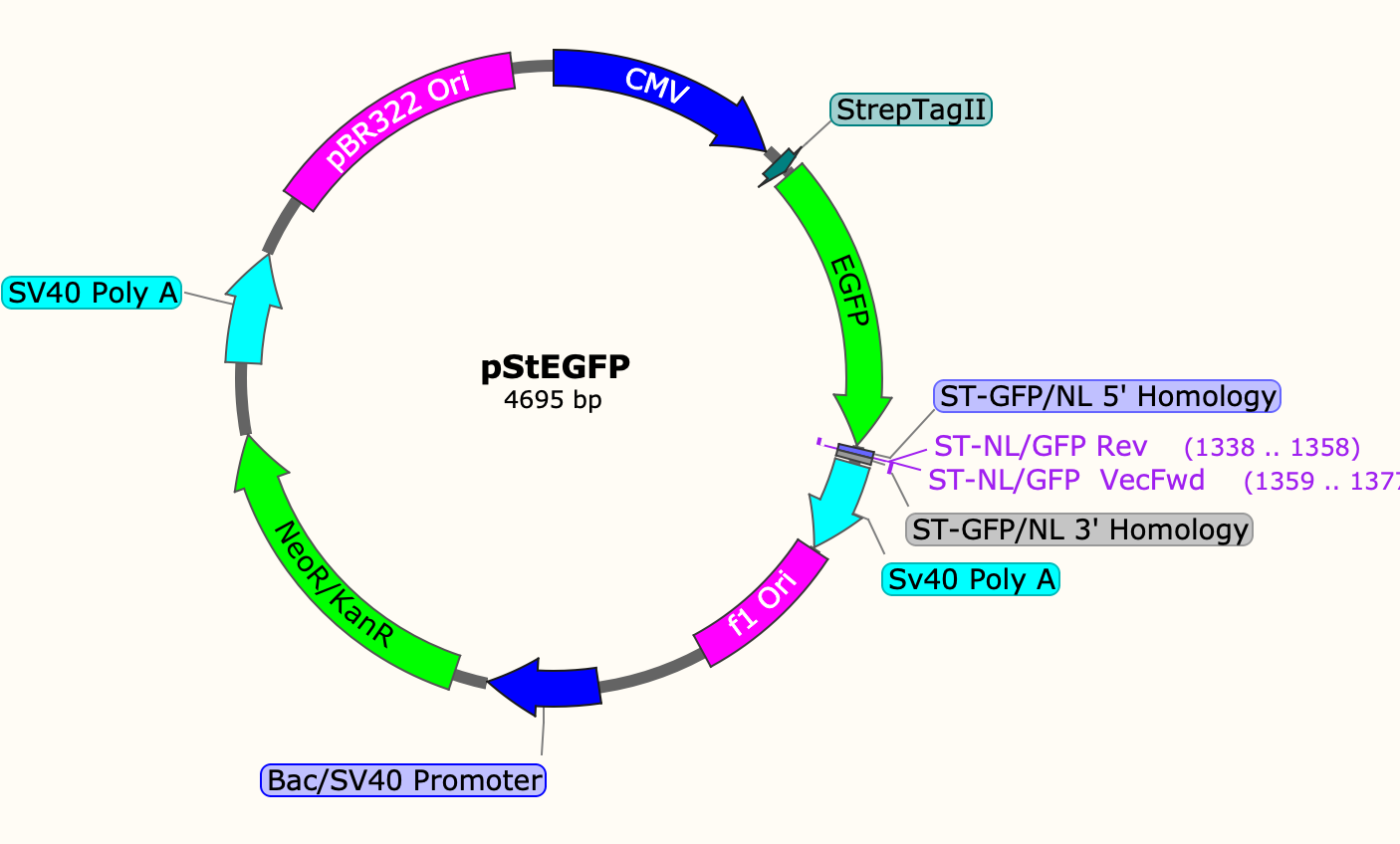

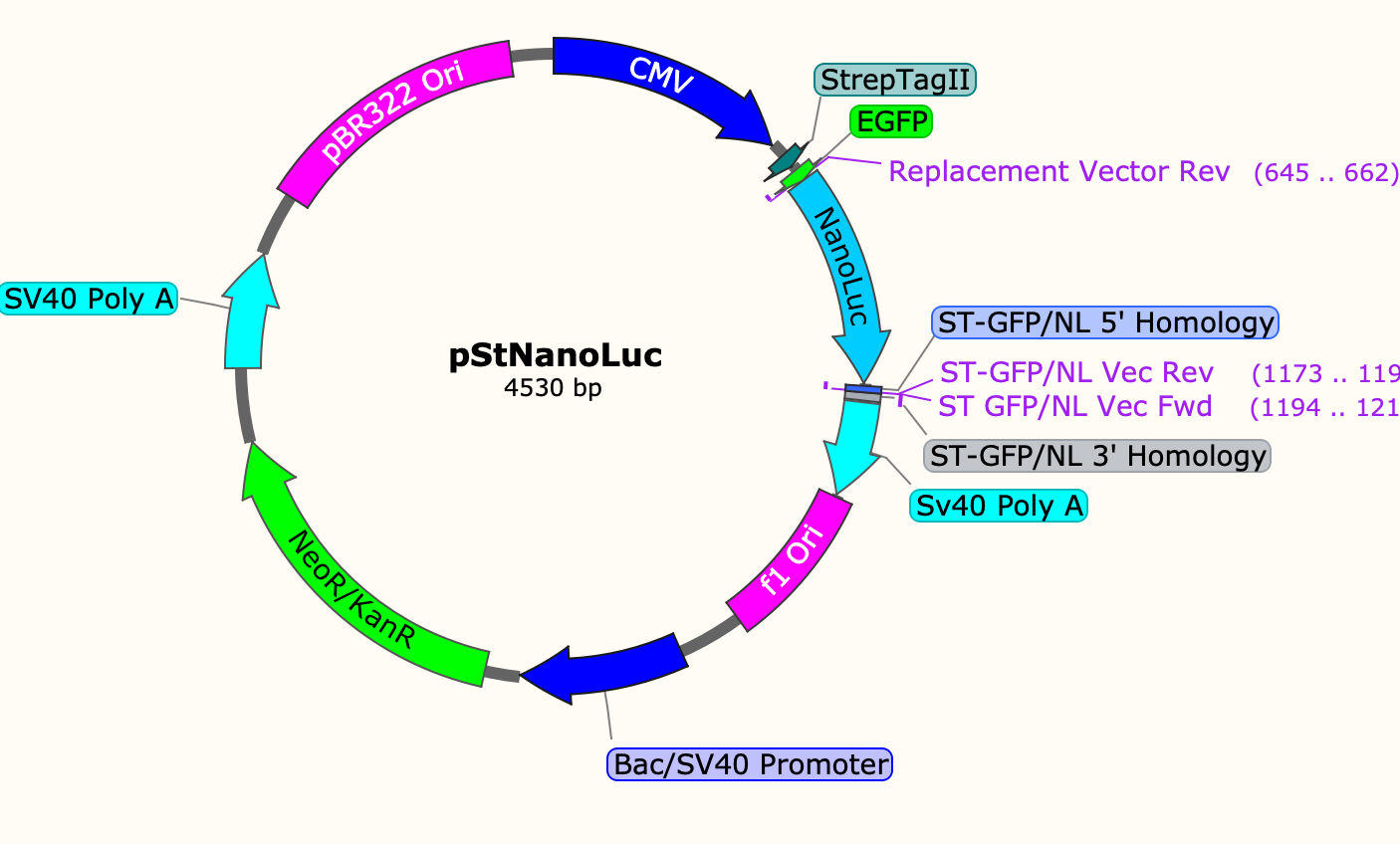


GFL/NLst Vec Fwd GTGAGCAAGGGCGAGGAG

GFL/NLst Vec Rev CATggtggcgaccggtg


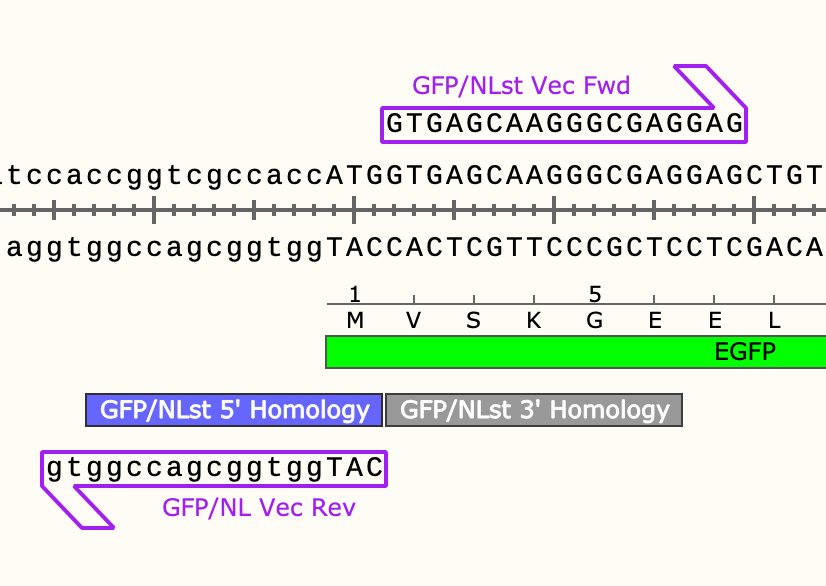


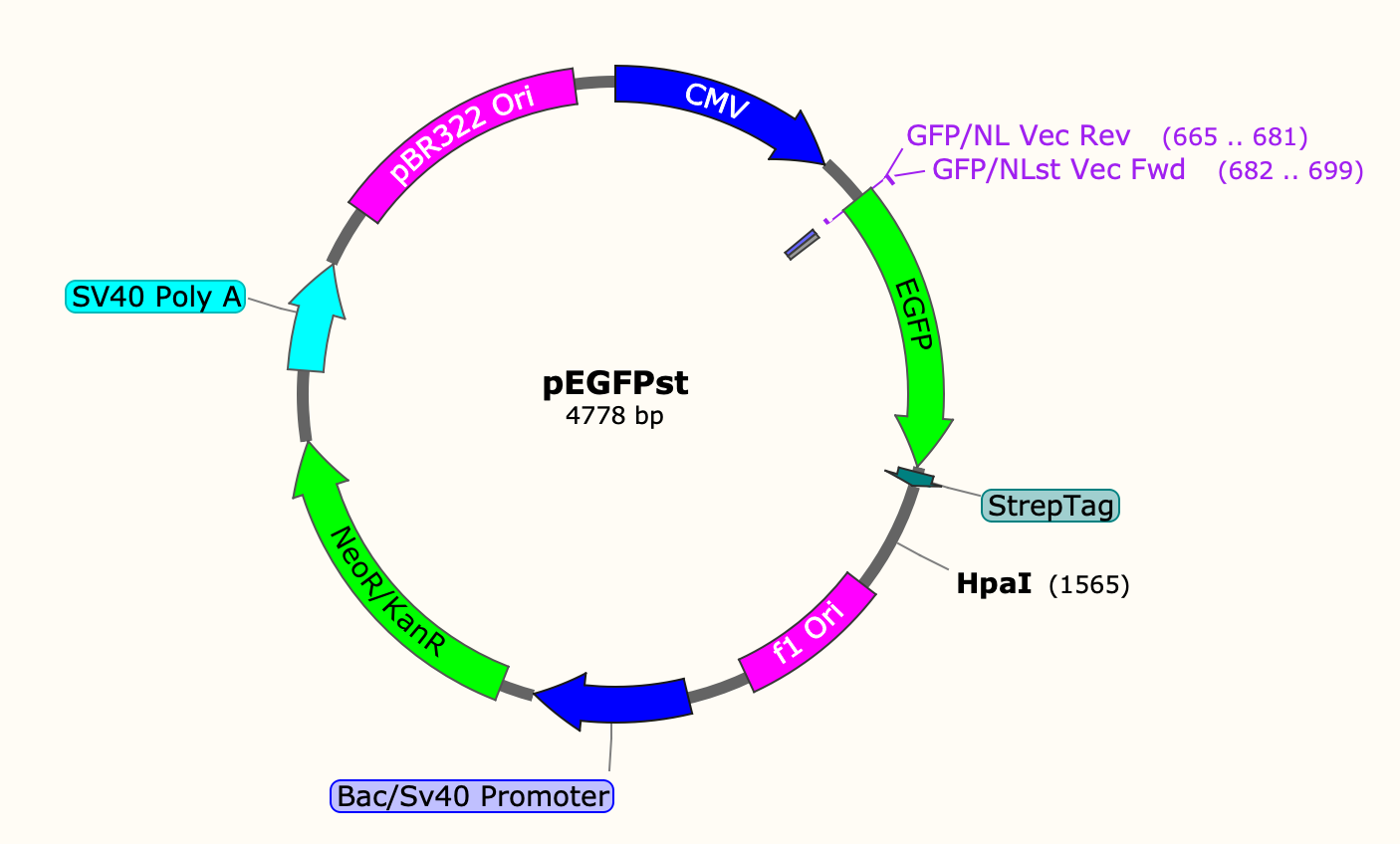

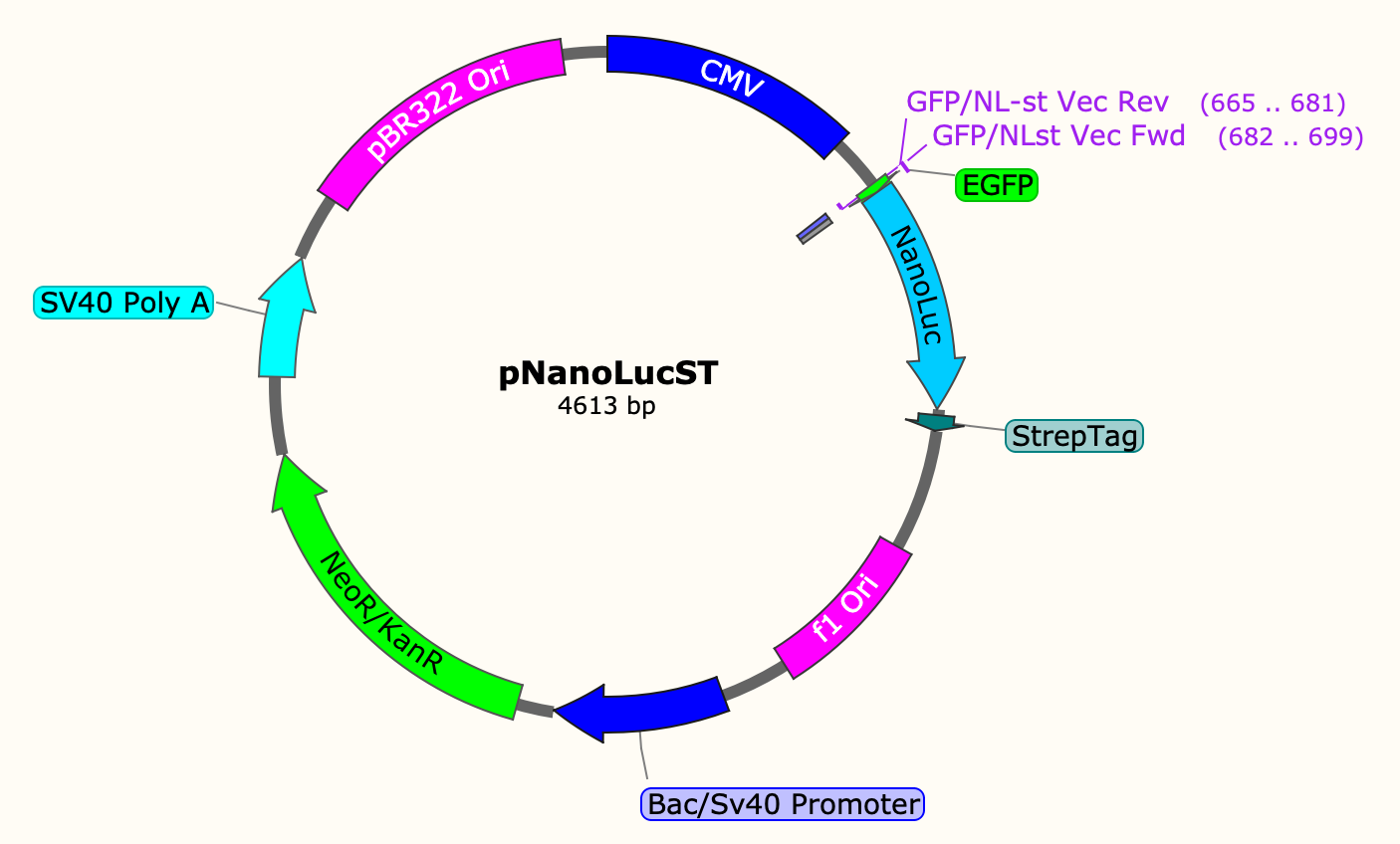


**Merlin Fusion Protein Amino Acid Sequences**

Merlin sequences are uncolored. Spacer sequences are in yellow. StrepTag II is in teal, EGFP is in green and NanoLuc is in light blue.

**Merlin-GFPst**

MAGAIASRMSFSSLKRKQPKTFTVRIVTMDAEMEFNCEMKWKGKDLFDLVCRTLGLRETWFFGLQYTIKDTVAWLKMDKKVLDHDVSKEEPVTFHFLAKFYPENAEEELVQEITQHLFFLQVKKQILDEKIYCPPEASVLLASYAVQAKYGDYDPSVHKRGFLAQEELLPKRVINLYQMTPEMWEERITAWYAEHRGRARDEAEMEYLKIAQDLEMYGVNYFAIRNKKGTELLLGVDALGLHIYDPENRLTPKISFPWNEIRNISYSDKEFTIKPLDKKIDVFKFNSSKLRVNKLILQLCIGNHDLFMRRRKADSLEVQQMKAQAREEKARKQMERQRLAREKQMREEAERTRDELERRLLQMKEEATMANEALMRSEETADLLAEKAQITEEEAKLLAQKAAEAEQEMQRIKATAIRTEEEKRLMEQKVLEAEVLALKMAEESERRAKEADQLKQDLQEAREAERRAKQKLLEIATKPTYPPMNPIPAPLPPDIPSFNLIGDSLSFDFKDTDMKRLSMEIEKEKVEYMEKSKHLQEQLNELKTEIEALKLKERETALDILHNENSDRGGSSKHNTIKKLTLQSAKSRVAFFEELGSTMVSKGEELFTGVVPILVELDGDVNGHKFSVSGEGEGDATYGKLTLKFICTTGKLPVPWPTLVTTLTYGVQCFSRYPDHMKQHDFFKSAMPEGYVQERTIFFKDDGNYKTRAEVKFEGDTLVNRIELKGIDFKEDGNILGHKLEYNYNSHNVYIMADKQKNGIKVNFKIRHNIEDGSVQLADHYQQNTPIGDGPVLLPDNHYLSTQSALSKDPNEKRDHMVLLEFVTAAGITLGMDELYKWSHPQFEK

**Merlin-NLst**

MAGAIASRMSFSSLKRKQPKTFTVRIVTMDAEMEFNCEMKWKGKDLFDLVCRTLGLRETWFFGLQYTIKDTVAWLKMDKKVLDHDVSKEEPVTFHFLAKFYPENAEEELVQEITQHLFFLQVKKQILDEKIYCPPEASVLLASYAVQAKYGDYDPSVHKRGFLAQEELLPKRVINLYQMTPEMWEERITAWYAEHRGRARDEAEMEYLKIAQDLEMYGVNYFAIRNKKGTELLLGVDALGLHIYDPENRLTPKISFPWNEIRNISYSDKEFTIKPLDKKIDVFKFNSSKLRVNKLILQLCIGNHDLFMRRRKADSLEVQQMKAQAREEKARKQMERQRLAREKQMREEAERTRDELERRLLQMKEEATMANEALMRSEETADLLAEKAQITEEEAKLLAQKAAEAEQEMQRIKATAIRTEEEKRLMEQKVLEAEVLALKMAEESERRAKEADQLKQDLQEAREAERRAKQKLLEIATKPTYPPMNPIPAPLPPDIPSFNLIGDSLSFDFKDTDMKRLSMEIEKEKVEYMEKSKHLQEQLNELKTEIEALKLKERETALDILHNENSDRGGSSKHNTIKKLTLQSAKSRVAFFEELGSTMVSKGEEVFTLEDFVGDWRQTAGYNLDQVLEQGGVSSLFQNLGVSVTPIQRIVLSGENGLKIDIHVIIPYEGLSGDQMGQIEKIFKVVYPVDDHHFKVILHYGTLVIDGVTPNMIDYFGRPYEGIAVFDGKKITVTGTLWNGNKIIDERLINPDGSLLFRVTINGVTGWRLCERILAGMDELYKWSHPQFEK*

**ST-GFP-Merlin**

MSAWSHPQFEKPVATMVSKGEELFTGVVPILVELDGDVNGHKFSVSGEGEGDATYGKLTLKFICTTGKLPVPWPTLVTTLTYGVQCFSRYPDHMKQHDFFKSAMPEGYVQERTIFFKDDGNYKTRAEVKFEGDTLVNRIELKGIDFKEDGNILGHKLEYNYNSHNVYIMADKQKNGIKVNFKIRHNIEDGSVQLADHYQQNTPIGDGPVLLPDNHYLSTQSALSKDPNEKRDHMVLLEFVTAAGITLGMDELYKMAGAIASRMSFSSLKRKQPKTFTVRIVTMDAEMEFNCEMKWKGKDLFDLVCRTLGLRETWFFGLQYTIKDTVAWLKMDKKVLDHDVSKEEPVTFHFLAKFYPENAEEELVQEITQHLFFLQVKKQILDEKIYCPPEASVLLASYAVQAKYGDYDPSVHKRGFLAQEELLPKRVINLYQMTPEMWEERITAWYAEHRGRARDEAEMEYLKIAQDLEMYGVNYFAIRNKKGTELLLGVDALGLHIYDPENRLTPKISFPWNEIRNISYSDKEFTIKPLDKKIDVFKFNSSKLRVNKLILQLCIGNHDLFMRRRKADSLEVQQMKAQAREEKARKQMERQRLAREKQMREEAERTRDELERRLLQMKEEATMANEALMRSEETADLLAEKAQITEEEAKLLAQKAAEAEQEMQRIKATAIRTEEEKRLMEQKVLEAEVLALKMAEESERRAKEADQLKQDLQEAREAERRAKQKLLEIATKPTYPPMNPIPAPLPPDIPSFNLIGDSLSFDFKDTDMKRLSMEIEKEKVEYMEKSKHLQEQLNELKTEIEALKLKERETALDILHNENSDRGGSSKHNTIKKLTLQSAKSRVAFFEEL

**ST-NL-Merlin**

MSAWSHPQFEKPVATMVSKGEEVFTLEDFVGDWRQTAGYNLDQVLEQGGVSSLFQNLGVSVTPIQRIVLSGENGLKIDIHVIIPYEGLSGDQMGQIEKIFKVVYPVDDHHFKVILHYGTLVIDGVTPNMIDYFGRPYEGIAVFDGKKITVTGTLWNGNKIIDERLINPDGSLLFRVTINGVTGWRLCERILAGMDELYKMAGAIASRMSFSSLKRKQPKTFTVRIVTMDAEMEFNCEMKWKGKDLFDLVCRTLGLRETWFFGLQYTIKDTVAWLKMDKKVLDHDVSKEEPVTFHFLAKFYPENAEEELVQEITQHLFFLQVKKQILDEKIYCPPEASVLLASYAVQAKYGDYDPSVHKRGFLAQEELLPKRVINLYQMTPEMWEERITAWYAEHRGRARDEAEMEYLKIAQDLEMYGVNYFAIRNKKGTELLLGVDALGLHIYDPENRLTPKISFPWNEIRNISYSDKEFTIKPLDKKIDVFKFNSSKLRVNKLILQLCIGNHDLFMRRRKADSLEVQQMKAQAREEKARKQMERQRLAREKQMREEAERTRDELERRLLQMKEEATMANEALMRSEETADLLAEKAQITEEEAKLLAQKAAEAEQEMQRIKATAIRTEEEKRLMEQKVLEAEVLALKMAEESERRAKEADQLKQDLQEAREAERRAKQKLLEIATKPTYPPMNPIPAPLPPDIPSFNLIGDSLSFDFKDTDMKRLSMEIEKEKVEYMEKSKHLQEQLNELKTEIEALKLKERETALDILHNENSDRGGSSKHNTIKKLTLQSAKSRVAFFEEL
