# Supplementary material for: Merlin tumor suppressor function is regulated by PIP2-mediated dimerization
Source: PLoS One. 2023 Feb 21;18(2):e0281876. doi: 10.1371/journal.pone.0281876 (PMC9942953; doi:10.1371/journal.pone.0281876)
Supplement: S1 Table — A table showing the numerical data shown in Figs 1D, 1F, 3B, 3C, 4A–4C. (DOCX) [file pone.0281876.s005.docx]

**Merlin Binding Data Tabular Summary**

| **Figure 1D** | **WT** | **FH** | **HC** | **H** | **GFP** |
| --- | --- | --- | --- | --- | --- |
| % Control | 100 | 65.1 | 8.10 | 3.02 | 0.36 |
| SD | 8.1 | 3.15 | 0.46 | 0.46 | 0.08 |
| n= | 3 | 3 | 3 | 3 | 3 |

| **Figure 1F** | **Mer-N**  **Mer-G** | **Mer-N**  **Mer-G** | **Mer-N**  **Mer-G** | **Mer-N**  **Mer-G** |
| --- | --- | --- | --- | --- |
| % Mer-N:Mer-G | 100 | 25 | 18 | 3 |
| SD | 5 | 2 | 0.3 | 0.22 |
| N= | 3 | 3 | 3 | 3 |

| **Figure 3B** | **WT** | **L64P** | **Δ39-121** | **L360P** | **L535P** | **6N** | **GFP** |
| --- | --- | --- | --- | --- | --- | --- | --- |
| % WT | 100 | 128 | 4 | 13 | 28 | 31 | 0 |
| SD | 4 | 5 | 0.1 | 0.1 | 2.1 | 5 | 0 |
| N= | 3 | 3 | 3 | 3 | 3 | 3 | 3 |

| **Figure 3C** | **WT** | **ΔN18** | **N20** | **S518A** | **S518D** | **AR** | **ΔEL** | **GFP** |
| --- | --- | --- | --- | --- | --- | --- | --- | --- |
| % WT | 100 | 33 | 0 | 205 | 56 | 9 | 206 | 0 |
| SD | 4 | 0.3 | 0 | 0.1 | 2.1 | 5 | 0 | 0 |
| N= | 3 | 3 | 3 | 3 | 3 | 3 | 3 | 3 |

| **Figure 4A** | **WT** | **S518A** | **S518D** | **AR** | **ΔEL** | **NL** |
| --- | --- | --- | --- | --- | --- | --- |
| % WT | 100 | 121 | 31 | 127 | 58 | 4 |
| SD | 16 | 32 | 12 | 42 | 15 | 5 |
| n= | 3 | 3 | 3 | 3 | 3 | 3 |

| **Figure 4B** | **WT** | **S518A** | **S518D** | **AR** | **ΔEL** | **NL** |
| --- | --- | --- | --- | --- | --- | --- |
| % WT | 100 | 178 | 30 | 14 | 294 | 1 |
| SD | 33 | 23 | 5 | 2 | 103 | 0 |
| n= | 3 | 3 | 3 | 3 | 3 | 3 |

| **Figure 4C** | **WT** | **S518A** | **S518D** | **AR** | **ΔEL** | **NL** |
| --- | --- | --- | --- | --- | --- | --- |
| % WT | 100 | 123 | 13 | 22 | 94 | 3 |
| SD | 17 | 1 | 2 | 6 | 41 | 0 |
| n= | 3 | 3 | 3 | 3 | 3 | 3 |

| **Figure 4D** | **WT** | **S518A** | **S518D** | **AR** | **ΔEL** | **NL** |
| --- | --- | --- | --- | --- | --- | --- |
| % WT | 100 | 162 | 64 | 14 | 139 | 4 |
| SD | 19 | 19 | 14 | 0 | 6 | 2 |
| n= | 3 | 3 | 3 | 3 | 3 | 3 |
